# Design of an Elliptic Curve Cryptography Processor for RFID Tag Chips

**DOI:** 10.3390/s141017883

**Published:** 2014-09-26

**Authors:** Zilong Liu, Dongsheng Liu, Xuecheng Zou, Hui Lin, Jian Cheng

**Affiliations:** 1 School of Optical and Electronic Information, Huazhong University of Science and Technology, Wuhan 430074, China; E-Mails: zilongliu@hust.edu.cn (Z.L.); estxczou@gmail.com (X.Z.); swordheartsmile@gmail.com (J.C.); 2 School of Science, Wuhan University of Technology, Wuhan 430074, China; E-Mail: linhuiwhut@163.com

**Keywords:** wireless sensor network, RFID, Elliptic Curve Cryptography (ECC), processor

## Abstract

Radio Frequency Identification (RFID) is an important technique for wireless sensor networks and the Internet of Things. Recently, considerable research has been performed in the combination of public key cryptography and RFID. In this paper, an efficient architecture of Elliptic Curve Cryptography (ECC) Processor for RFID tag chip is presented. We adopt a new inversion algorithm which requires fewer registers to store variables than the traditional schemes. A new method for coordinate swapping is proposed, which can reduce the complexity of the controller and shorten the time of iterative calculation effectively. A modified circular shift register architecture is presented in this paper, which is an effective way to reduce the area of register files. Clock gating and asynchronous counter are exploited to reduce the power consumption. The simulation and synthesis results show that the time needed for one elliptic curve scalar point multiplication over *GF*(2^163^) is 176.7 K clock cycles and the gate area is 13.8 K with UMC 0.13 μm Complementary Metal Oxide Semiconductor (CMOS) technology. Moreover, the low power and low cost consumption make the Elliptic Curve Cryptography Processor (ECP) a prospective candidate for application in the RFID tag chip.

## 1. Introduction

Radio frequency identification (RFID) is an auto identification technology. Nowadays, it is widely used for identification control, chain management, wireless sensor networks (WSNs) and other applications. With the rapid development of the Internet of Things (IOT) and WSN, the demand on security-related RFID systems has grown fast [1]. These RFID applications require low-power and low-cost implementations of security mechanisms.

Recently, symmetric key cryptography, such as Advanced Encryption Standard (AES), has been suggested that it might not be preferable for RFID systems, since the number of RFID tags can be very large in WSNs and there is a potential risk involved in storing numerous symmetric keys. To satisfy security and system requirements, it is proved that a suitable public key cryptography scheme is necessary. Due to the fact that the traditional public key cryptography adds high overhead to the RFID tag chip, it has been considered to be unsuitable for a long time. For instance, passive RFID tags obtain energy from radio frequency signals, the power supply is limited. Therefore, these tags cannot utilize the energy-demanding cryptographic algorithms, such as the well-known RSA cryptography. Nevertheless, Elliptic Curve Cryptography (ECC), proposed by Koblitz [2], has been employed in many applications recently due to its numerous advantages over traditional public key cryptography schemes. The main advantage is that in utilizing the smaller key sizes, ECC can offer the similar security level as RSA [3]. For example, the security of 163-bit ECC is considered equivalent to 1024-bits RSA [4]. This feature makes it highly suited for implementation in RFID tag chips and being used in WSNs extensively.

The security of ECC is based on the difficulty of elliptic curve discrete logarithm problem (ECDLP) and the underlying operation in the elliptic curve cryptosystems is scalar point multiplication. The point multiplication can be performed by finite field arithmetic computations such as field addition, field multiplication, field squaring, and inversion. A number of hardware implementations for elliptic curve cryptography have been suggested in literatures, but only a few of them are aimed for RFID. Most implementations focus on speed and are based on the field-programmable gate array (FPGA) technology [5–9].

There are several implementations of scalar point multiplication in the literatures targeting RFID tag chips [10–13]. These implementations are different from coordinate systems (e.g., affine, projective, and mixed), basis (e.g., polynomial basis, normal basis), curves, architectures or algorithms. Most of these implementation efforts are concentrated on reducing the register number of elliptic curve cryptography processor without considering the practical applications, such as the transaction time. Generally, the total transaction time of RFID is required less than 300 ms [14]. However, most papers for RFID show that the time consumption to finish one point multiplication is over or close to 300 ms. Some papers just deliver a single ECC processor without involving the interfaces for other modules in a RFID tag chip, such as ROM, RAM and system controller. Low power consumption is an essential requirement for passive RFID tag chips. However, most related papers only focus on how to reduce the area of ECC processor for RFID tag chips. It has been ignored for a long time to reduce the power consumption with various low power techniques.

In this paper, we present an efficient implementation of Elliptic Curve Cryptography Processor (ECP) targeting RFID tag chips. First of all, the architecture of the RFID tag chip with ECP is described. Later on, we analyze different algorithms for scalar point multiplication and inversion, which are underlying operations in the elliptic curve cryptosystem. A new inversion algorithm is adopted and it needs fewer registers to store variables than the traditional methods. Then, we design a compact and efficient modular arithmetic logic unit (MALU). The modified circular shift register architecture is presented in this paper. We optimize the primary architecture and it shows an effective way to reduce the area of register files. In this paper, a new method of coordinates swapping is proposed which can reduce the complexity of the controller and shorten the time of iterative calculation effectively. In order to achieve low power consumption, clock gating and asynchronous counters are extensively used in our design. Finally, we perform an evaluation of our design and make comparisons with previous works.

The proposed design requires less clock cycles to finish one scalar point multiplication. The total time spent on scalar point multiplication is nearly 176.7 K clock cycles. The compact ECP needs only the area of 0.12 mm^2^ with UMC 0.13 μm technology and the power consumption is 20.1 μW at the clock frequency of 847.5 KHz. All of these characteristics make the ECP design very attractive for RFID tag chips.

The rest of the paper is organized as follows: Section 2 presents the architecture of the RFID tag chip with our proposed ECP, and in Section 3, the ECC algorithms are analyzed and optimized. Section 4 presents the implementation design for the different modular arithmetic logic units. In Sections 5 and 6, the modified register array and the structure of ECP command controller are presented. In Section 7, the low power strategies are presented. Result analysis and comparison are carried out in Section 8. Finally, we conclude this paper in Section 9.

## 2. The Architecture of the RFID Tag Chip with ECP

**Figure 1.**
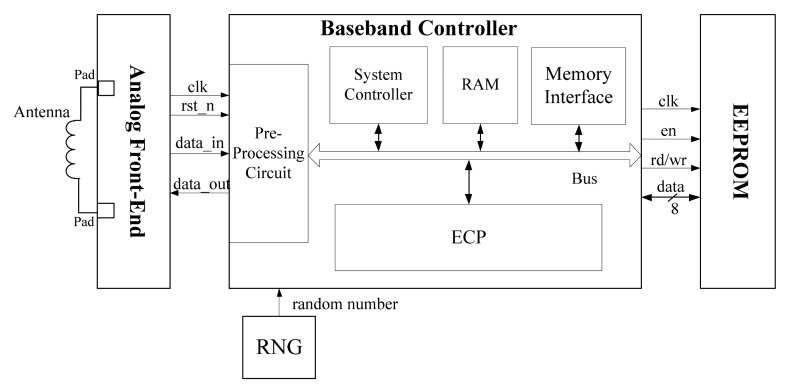
The structure of Radio Frequency Identification (RFID) tag with an Elliptic Curve Cryptography Processor (ECP).

A typical ECP-embedded RFID tag chip can be divided into four parts [15], including Analog Front End (AFE), Random Number Generator (RNG), EEPROM and Digital Baseband Controller, as illustrated in Figure 1. AFE accomplishes the detailed functions of physical layer according to the RFID protocol, including carrier signal demodulation, modulation, power supply, clock generation, and reset signal generation. Random numbers generated from RNG will be used in elliptic curve digital signature algorithm (ECDSA). RNG can make sure the randomness property of each authentication so that the data in the authentication is unpredictable. EEPROM is used for storing private or public information, such as the private key, base point of elliptic curve (EC) and the EC equation parameters. Baseband Controller, utilizing the streamline bus structure, integrates the pre-processing circuit, RAM block, system controller, memory interface and ECP into one unit.

After the AFE demodulates a frame sent by the reader, then the pre-processing circuit will check the validation of the frame and extract the useful information from the frame. If the frame is legitimate, the RAM block will store the frame data into the memory arrays through the bus. When the phase of data receiving is over, the system controller will read the related information through the bus and load them into the ECP unit for further calculation. When the initialization of EC parameters has been fulfilled, the system controller will inform the ECP to start related calculation and wait for the signals from the ECP before entering the responding phase.

In this paper, the structure of the ECP is suitable for Elliptic Curves (ECs) in binary extension field of *GF*(2*^m^*), which means the value of *m* can be any legitimate value. However, for better discussion, *m* is set to 163, and the reduction polynomial is shown as follows:

(1) $$F\left(x\right)=x^{163}+x^{7}+x^{6}+x^{3}+1$$

**Figure 2.**
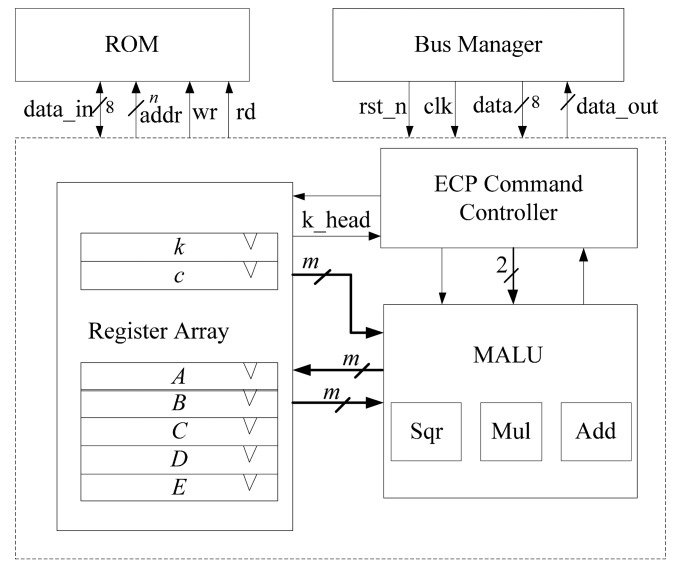
Structure of ECP.

As shown in Figure 2, we propose a structure of the ECP, composed of register array, MALU and ECP command controller. The ECP performs the scalar point multiplication, inversion and any other ECC calculations and returns the results of the calculations via the bus. In our implementation, the ECP loads the private key *k*, ECs parameter *c* and base point *P*(x,y) into register array from ROM and executes the scalar point multiplication or other operations. After finishing the calculation, it outputs the results for further use.

## 3. Scalar Point Multiplication

### 3.1. Arithmetic Analysis

Scalar point multiplication, *Q* = *k*·*P*, is the underlying operation in the elliptic curve cryptosystems. *P* is the base point of ECs and *k* is a scalar used as private key. The resultant point *Q* will be used as a public key. According to ECDLP, if *k* is significantly large then it is very hard to retrieve *k* when the values of *P* and *Q* are given. The scalar point multiplication can be executed by point additions and point doublings, both of which involve many basic field arithmetic operations. In this paper, the EC is set as a generic Koblitz curve with the form of Equation (2), which is widely used in ECC, and the basic arithmetic operations are performed in the Galois field (*GF*). The GFs are either prime field *GF*(*p*) or extension binary field *GF*(2*^m^*). The *GF*(2*^m^*) design is easier for hardware implementation and is adopted in this paper.

(2) $$y^{2}+xy=x^{3}+ax^{2}+b$$

There are two commonly used algorithms for scalar point multiplication, namely Binary Method [16] and Montgomery Ladder Method [17]. Binary Method is a basic scalar point multiplication method, also called *double and add* method, as shown in Algorithm 1. The scalar point multiplication iterates through every bit of *k*. In each iteration, the point doubling is performed. When the particular bit of *k* is one, the point addition is also performed. It means that the execution time of one scalar point multiplication is correlated to the hamming weight of the key *k*, and then the Simple Power Analysis (SPA) attacks become a threat to reveal the key value through recording power traces over time.


**Algorithm 1.** Binary Method for scalar point multiplication.**Input :**
*k and P*, *k* = (*k_m_*_−1_,*k_m_*_−2_,…*k*_0_)_2_, *k_i_*∈{0,1}**Output :**
*Q* = *k*·*P*1 : *set Q*←02 : ***for** i from m*−1*to* 0 ***do***3 :   **Q←2*Q*4 :   ***if** k_i_* =1 ***then** Q*←*Q* + *P*5 : *return Q*

Montgomery ladder method is one of the most commonly used algorithms to perform scalar point multiplication. The advantage of this method is that only the *x*-coordinate is used for point doubling and point addition in affine coordinates. Hence, the number of field operations and register variables can be reduced in each iteration. However, in affine coordinates, two inversion operations are needed in each iteration. As a result, at least 2 m field inversions should be performed to finish one scalar point multiplication, as shown in Algorithm 2, which leads to huge time consumption and is conflict with RFID real-time requirement. Therefore, we introduce the López-Dahab projective coordinate [18].


**Algorithm 2.** Montgomery Ladder Method in affine coordinates.**Input :**
*P*,*k*, *where P* = (*x,y*)∈ *GF*(2*^m^*), *k* = [*k_t_*_−1_ ⋯ *k*_1_,*k*_0_]∈ *Z*^+^**Output :**
*Q* = *kP* = (*x*_1_,*y*_1_)1 : *if k* = 0 *or x* = 0 *then return Q* = (0,0) *and stop*2 : *set x*_1_←*x*, *x*_2_←*x*^2^+*b*/*x*^2^3 : *for i* = *t*−2 *decreto* 0 *do* 
$l=\frac{x_{1}}{\left(x_{1}+x_{2}\right)}$ *if k_i_*=1, *set*
$x_{1}\leftarrow x+l^{2}+l,x_{2}\leftarrow x_{2}^{2}+\frac{b}{x_{2}^{2}}$ *else set*
$x_{2}\leftarrow x+l^{2}+l,x_{1}\leftarrow x_{1}^{2}+\frac{b}{x_{1}^{2}}$4 : *set r*_1_←*x*_1_+*x*,*r*_2_←*x*_2_+*x*5 : *set*
$y_{1}\leftarrow \frac{r_{1}(r_{1}r_{2}+x^{2}+y)}{x}+y$6 : *return Q*(*x*_1_,*y*_1_)
**Algorithm 3.** Montgomery ladder algorithm in López-Dahab coordinates.**Input :**
*P*, *k*,*where P* = (*x*,*y*)∈*GF*(2*^m^*),*k* = [*k_t_*_−1_⋯*k*_1_,*k*_0_]∈*Z*^+^**Output :**
*Q* = *kP* = (*x*_1_,*y*_1_)1: *if k* = 0 *or x* = 0 *then return Q* = (0,0) *and stop*2: *set X*_1_←*x*,*Z*_1_←1,*X*_2_←*x*^4^+*b*,*Z*_2_←*x*^2^3: *for i*=*t*−2 *decreto* 0 *do*  *if k_i_* = 1, *set* (*X*_1_,*Z*_1_) = *Madd*(*X*_1_,*Z*_1_,*X*_2_,*Z*_2_)        (*X***_2_**,*Z***_2_**)= *Mdouble*(*X*_2_, *Z*_2_)  *else set* (*X*_2_,*Z*_2_)=*Madd*(*X*_2_,*Z*_2_,*X*_1_,*Z*_1_)        (*X*_1_,*Z*_1_)=*Mdouble*(*X*_1_,*Z*_1_)4 : *set Q*(*x*_1_,*y*_1_)=*Mxy*(*X*_1_,*Z*_1_,*X*_2_,*Z*_2_)5 : *return Q*(*x*_1_,*y*_1_)*Note* :
$\text{Madd}=\{\begin{array}{c} X_{\text{add}}=x\times Z_{\text{add}}+(X_{1}\times Z_{2})\times (X_{2}\times Z_{1})\\ Z_{\text{add}}={\left(X_{1}\times Z_{2}+X_{2}\times Z_{1}\right)}^{2} \end{array}$
$\text{Mdouble}=\{\begin{array}{c} X_{\text{double}}=X_{2}^{4}+b\times Z_{2}^{4}\\ Z_{\text{double}}={\left(X_{2}\times Z_{2}\right)}^{2} \end{array}$
$Mxy=\{\begin{array}{c} x_{1}=\frac{X_{1}}{Z_{1}}\\ y_{1}=y+\frac{X_{1}\times (X_{1}\times X_{2}+x\times X_{1}\times Z_{2}+x\times X_{2}\times Z_{1}+y\times Z_{1}\times Z_{2})}{x\times Z_{2}\times Z_{1}^{2}} \end{array}$

In projective coordinates, X-coordinate and Z-coordinate are used in each iteration, but the inversion operation is avoided. Only one inversion operation is performed in the conversion from projective coordinates to affine coordinates, as shown in Algorithm 3. It will dramatically reduce the time cost of the calculation. In the affine coordinates, because the inversion operation is performed in each iteration, 6 *m*-bit register variables are needed in the calculation of each iteration, which is the same number of *m*-bit registers as the method in the projective coordinates. Compared to Binary method, Montgomery ladder method performs point addition and point doubling in each iteration regardless of the value of *k_i_*. It offers intrinsic protection against SPA and Timing Analysis (TA) [19].

In general, the scalar point multiplication can be divided into 4 basic computing elements in *GF*(2*^m^*), namely multiplication, addition, inversion and squaring. The inversion operation can be performed by multiplication and squaring. In this paper, we combine the adder, squarer and multiplier into one design unit called MALU.

### 3.2. Inversion Algorithm

In the end of Algorithm 3, Mxy(*X_1_, Z_1_, X_2_, Z_2_*) means the conversion from projective coordinates to affine coordinates. It requires only one inversion operation in Algorithm 3. As inversion is a complicated operation, the *Fermat's Little Theorem* provides a simple method to perform inversion as follows [20]:

(3) $$A^{-1}=A^{2^{m}-2}=A^{2(1+2+\ldots +2^{m-2})}$$

According to Equation (3), in the binary extension field of *GF*(2^163^), a total of 161 multiplications and 162 squarings are needed in one inversion operation. The previous Itoh-Tsujii (IT) algorithm is an effective scheme to reduce the number of multiplication operation in one inversion [21]. Algorithm 4 shows the inversion operation with the IT method. It is based on the following decomposition:

(4) $$1+2+2^{2}+\ldots 2^{161}=(2\times (2\times (1+2)(1+2^{2})+1)\times (1+2^{5})\times (1+2^{10})\times (1+2^{20})\times (1+2^{40})+1)\times (1+2^{81})$$

**Algorithm 4.** Inversion with IT method over *GF*(2^163^).**Input :**
*A*∈*GF*(2^163^)**Output :**
*T* =*A*^−1^=*A*^2^*^m^*^−2^ where *m* =1631: *B* ← *A*^2^2: *T* ← *B*×*A*,3: *B* ←*T*^2^2^^4: *T* ← *B*×*T*,5: *B* ←*T*^2^6: *T* ← *B*×*A*,7: *B* ←*T*^2^5^^8: *T* ← *B*×*T*,9: *B* ←*T*^2^10^^10: *B* ← *B*×*T*,11: *B* ←*T*^2^20^^12: *T* ← *B*×*T*,13: *B* ←*T*^2^40^^14: *T* ← *B*×*T*,15: *B* ← *T*^2^16: *T* ← *B*×*A*,17: *B* ← *T*^2^81^^18: *T* ← *B*×*T*,19: *T* ← *T*^2^

Recently, a new Dimitrov-Järvinen (DJ) algorithm for inversion was introduced [22]. The new DJ method is based on the ingenious decomposition as follows [23]:

(5) $$1+2+2^{2}+\ldots 2^{161}=(1+2+2^{2})\times (1+2^{3}+2^{6})\times (1+2^{9}+2^{18})\times (1+2^{27}+2^{54})\times (1+2^{81})$$

From Equations (4) and (5), in the binary extension field of *GF*(2^163^), IT method and DJ method both require 9 multiplications and 162 squarings. In the implementation of these two methods, IT method needs three 163-bit register variables, but DJ method only needs two 163-bits register variables. Our design has implemented the DJ method in projective coordinate system with only one inversion operation. The inversion algorithm with DJ method is shown in Algorithm 5.


**Algorithm 5.** Inversion with the Dimitrov-Järvinen (DJ) method over *GF*(2^163^).**Input :**
*A*∈*GF*(2^163^)**Output :***T* =*A*^−1^=*A*^2^m^−2^ where *m* =1631: *A* ← *A*^2^2: *T* ← *A*^2^,3: *A* ← *A*×*T*4: *T* ← *T*^2^,5: *A* ← *A*×*T*6: *T* ← *A*^2^3^^,7: *A* ← *A*×*T*8: *T* ← *T*^2^3^^,9: *A* ← *A*× *T*10: *T* ← *A*^2^9^^,11: *A* ← *A*×*T*12: *T* ← *T*^2^9^^,13: *A* ← *A*×*T*14:*T* ← *A*^2^27^^,15: *A* ← *A*×*T*16 :*T* ← *T*^2^27^^,17 : *A* ← *A*×*T*18:*T* ← *A*^2^81^^,19:*T* ← *A*×*T*

## 4. Modular Arithmetic Logic Unit (MALU)

**Figure 3.**
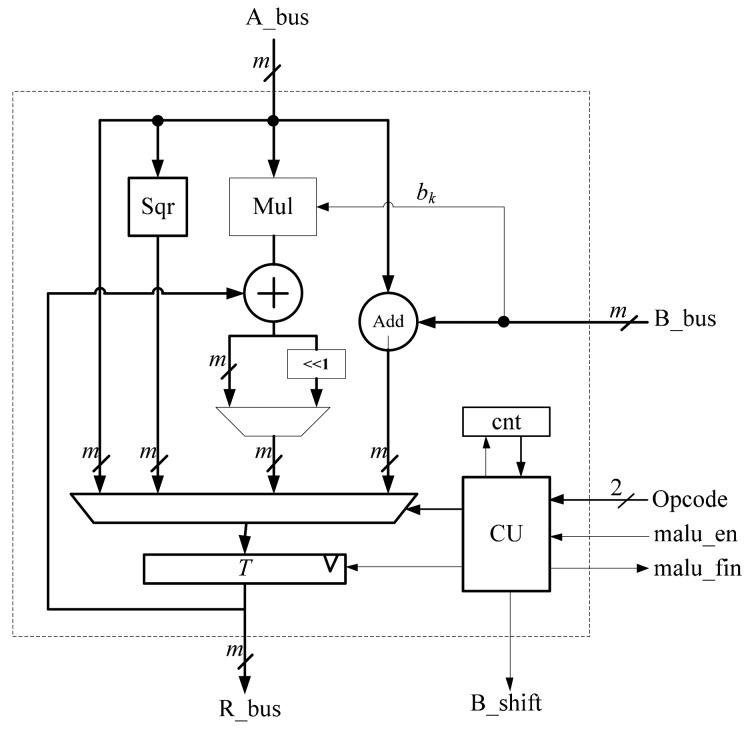
Structure of the modular arithmetic logic unit (MALU).

The MALU shown in Figure 3 performs the *GF*(2*^m^*) field operations, namely the multiplication, addition, squaring and inversion. The inversion operation can be performed by multiplications and squarings. The MALU contains 5 units, an adder unit, multiplier unit, squarer unit, controller unit and register *T*. The MALU is designed to execute 4 basic commands, namely ADD, SQR, MUL and INV. The operands for these commands are stored in the register array. *Opcode* is used to select which operation to be performed. When the signal of *malu_en* is valid and the MALU gets the input operands through the bus of *A_bus* and *B_bus*, the MALU will make proper operation according to the value of *Opcode*.

For addition, the field addition operation adds A and B, provided by *A_bus* and *B_bus*. It can be simply obtained by a bit-wise XOR operation. For squaring, the field squaring operation needs only one operand provided by *A_bus*. Addition and squaring operations can be efficiently performed in the MALU with a latency of one clock cycle.

Multiplication is more complex. By using the bit-serial multiplier, one operand is provided by *A_bus*. The multiplier requires the most significant bit (MSB) *b_k_* of *B_bus* for the multiplicand. The signal *B_shift* is used for multiplication and is provided to *Reg_B* in the register array. In the field of *GF*(2*^m^*), when the multiplication is performed, *B_shift* will be valid for *m* clock cycles, and the register *Reg_B* will shift to the left *m* times. The counter in the MALU is used to count the number of clock cycle for multiplication. It will take *m* clock cycles to finish one multiplication operation with the bit-serial multiplier.

After each command is executed, the MALU will provide a signal called *malu_fin* to inform up-level ECP for further computing. The register *T* serves as a medium for restoring the results of 4 basic commands and is responsible for exchanging data with register *Reg_A* through *R_bus*.

### 4.1. Adder Unit

For two elements, *A*=(*a_m_*_–1_,*a_m_*_–2_,…,*a*_0_)∈*GF*(2*^m^*) and *B*=(*b_m_*_–1_,*b_m_*_–2_,…,*b*_0_)∈*GF*(2*^m^*), field addition in the binary extension field of *GF*(2*^m^*) can be simply obtained by a bit-wise XOR addition operation as $A+B=\sum_{i=0}^{m-1}\left(a_{i}⊕b_{i}\right)x^{i}$. Therefore, the Adder Unit is implemented in our design using 163 XOR gates with one clock cycle output latency.

### 4.2. Squarer Unit

We represent *A*(*x*)∈*GF*(2*^m^*) in polynomial basis as follows:

(6) $$\begin{array}{cc} A(x)=\sum_{i=0}^{m-1}a_{i}x^{i}, & a_{i}\in GF(2) \end{array}$$

Squaring in *GF*(2*^m^*) can be calculated as follows:

(7) $$C(x)=A^{2}(x)=a_{m-1}x^{2m-2}+\cdot \cdot \cdot +a_{2}x^{4}+a_{1}x^{2}+a_{0}\mathrm{mod}F(x)$$

In the field of *GF*(2^163^), *m*=163, the field arithmetic is implemented as polynomial arithmetic modulo *F*(*x*). Using the equivalence in Equation (8), it can reduce the double-sized result with the reduction polynomial.

(8) $$x^{163}=x^{7}+x^{6}+x^{3}+1$$

From Equations (7) and (8), we can get the results as follows:

$$\begin{array}{l} c_{162}x^{162}=a_{81}x^{162}+a_{159}x^{162}+a_{161}x^{162}\\ c_{161}x^{161}=a_{159}x^{161}+a_{162}x^{161}\\ \cdots \\ c_{4}x^{4}=a_{2}x^{4}+a_{82}x^{4}+a_{160}x^{4}\\ c_{3}x^{3}=a_{83}x^{3}+a_{160}x^{3}+a_{161}x^{3}\\ c_{2}x^{2}=a_{1}x^{2}+a_{161}x^{2}\\ c_{1}x^{1}=a_{82}x^{1}+a_{160}x^{1}+a_{162}x^{1}\\ c_{0}=a_{0}+a_{160} \end{array}$$

**Figure 4.**
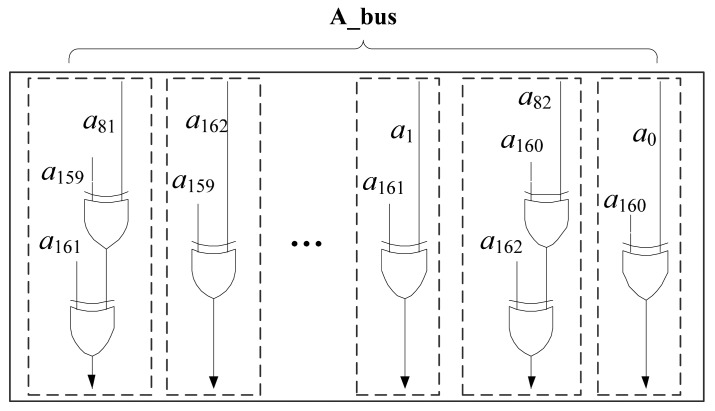
Squaring Circuit in *GF*(2^163^).

Therefore, without huge area requirements, squaring in *GF*(2*^m^*) can be efficiently implemented as a combinational logic circuit to output the result in one clock cycle. There are total 247 XOR gates in the squaring circuit. The operand comes from *A_bus* and the result can be saved in register *T*. The squarer unit is shown in Figure 4.

### 4.3. Multiplier Unit

**Figure 5.**
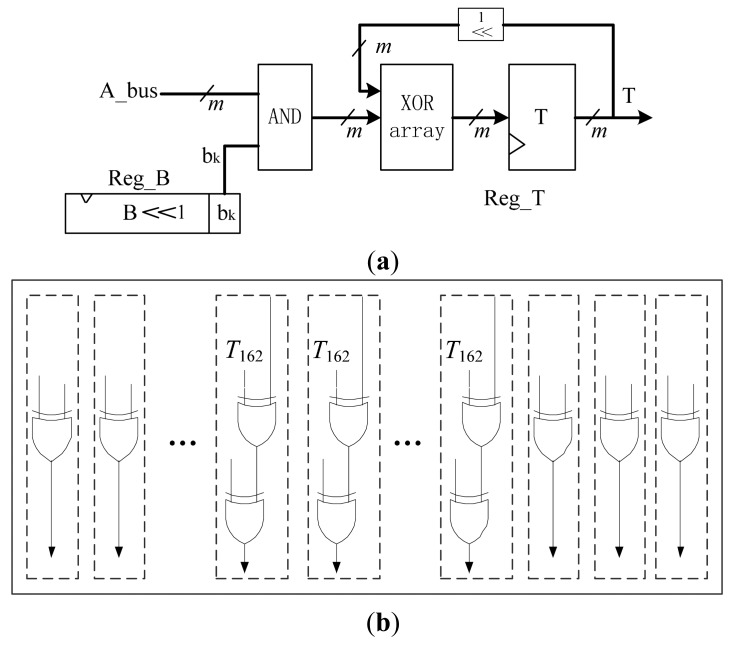
(**a**) The architecture of bit-serial multiplier Circuit in *GF*(2*^163^*); (**b**) The XOR array for multiplier.

The multiplier unit is the biggest component in the MALU. As the low-power and low-cost requirements of RFID, it is necessary to optimize the design of multiplier unit. Bit-serial multipliers are proved to be the most efficient scheme that can reduce area consumption and maintain good performance [11]. It will take *m* clock cycles to finish one multiplication operation. We implement the Bit-serial multipliers as Algorithm 6, and the structure is shown in Figure 5. The operand *A* can be enabled onto the *A_bus*, directly from the Register Array. The individual bit of *b_i_* comes from the MSB of *B_bus* which is directly from a 163-bits cyclic shift-register *Reg_B* in the Register Array. The XOR array is based on the reduction polynomial and is composed of 166 XOR gates. Therefore, there are total 163 AND gates, 166 XOR gates and a 163-bits shifter-register *T* in the bit-serial multiplier circuit. The register *T* is also used to store the results of addition and squaring.


**Algorithm 6.** Bit-serial multiplication in the field of *GF*(2^163^).**Input :**
$A(x)=\sum_{i=0}^{m-1}a_{i}x^{i}$, 
$B(x)=\sum_{i=0}^{m-1}b_{i}x^{i}$, *where a_i_,b_i_* ∈ {0,1}**Output :**
*T*
**=**
*A*·*B* mod 
$F(x)=\sum_{i=0}^{m-1}c_{i}x^{i}$, *where c* ∈ {0,1}1 : *T*←02 : **for**
*i* = *m*−1 *down to* 0 **do**  *T*←*b_i_*·*A*(*x*) + *T*·*x* + *t_m_*_−1_·*F*(*x*)3 : **Return**
*T*

## 5. Register Array

**Figure 6.**
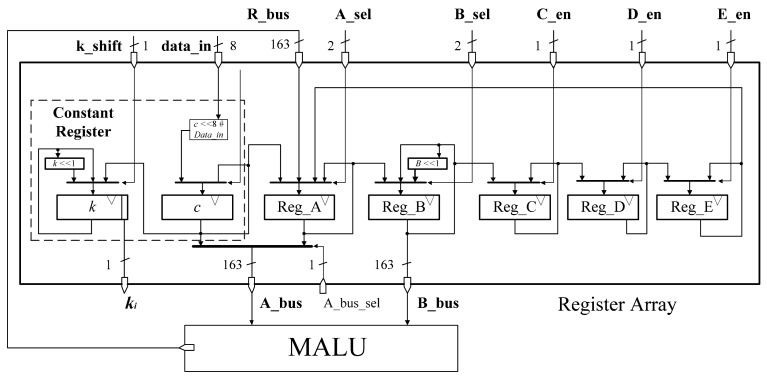
Register array file architecture.

The architecture of the register array file is shown in Figure 6. This circular shift register architecture has been proved to be an effective method to reduce the area consumption of register files [10]. We modify the primary architecture and make it fit for our whole design. In [10], the calculation that converting projective coordinates to affine coordinates is not involved and the register *k* is set in their controller module. Therefore, there are only six registers needed in their register file. In our register file, there are seven registers including register *k*. Register *k* and register *c* are the constant registers. Reg_A, Reg_B, Reg_C, Reg_D, Reg_E are used to store temporary variables of the calculation. Register *c* has an 8-bit I/O through which data coming from external ROM or RAM can be loaded and stored. These external data can be private key *k*, ECs parameter *c*, or the coordinates of base point *P*(x,y). When the ECP needs some constant parameters for calculation, register *c* will load those parameters. After the private key *k* is loaded into register *c* from the external ROM, register *k* will load the key value from register *c* immediately. After finishing the data preparation of part 2 in Algorithm 3, the register *k* can be shifted by one bit to the left according to the iteration calculation of part 3 in Algorithm 3 and provides the *k_i_* value for ECP command controller.

The whole register array is a circular shift register file. Each register is independently controlled by ECP command controller for efficient management. Furthermore, Reg_B is a circular shift register which can be shifted by one bit to the left. The data in Reg_A and Reg_B will input to MALU through *A_bus* and *B_bus* for the calculation based on *opcode*. Reg_C, Reg_D, Reg_E can only be updated by the preceding register, as the original scheme. To reduce the power consumption, clock gating, as a low power strategy, is applied to the circular shift register. Compared to the primary architecture, we integrate the register of private key *k* and the register of constant *c* into the register array. It makes our ECP become more compact and efficient.

## 6. ECP Command Controller

**Figure 7.**
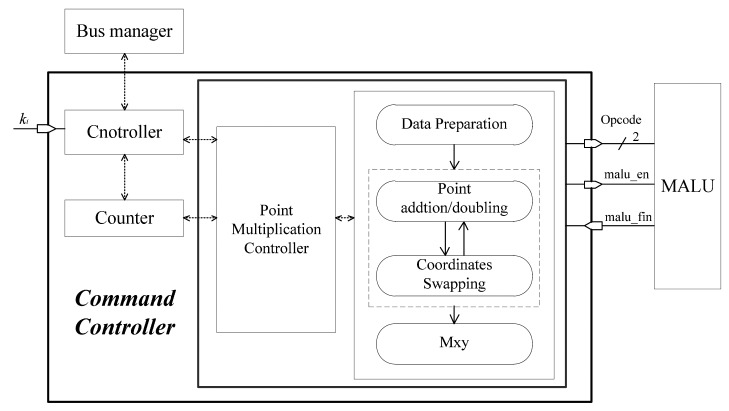
ECP Command Controller.

**Figure 8.**
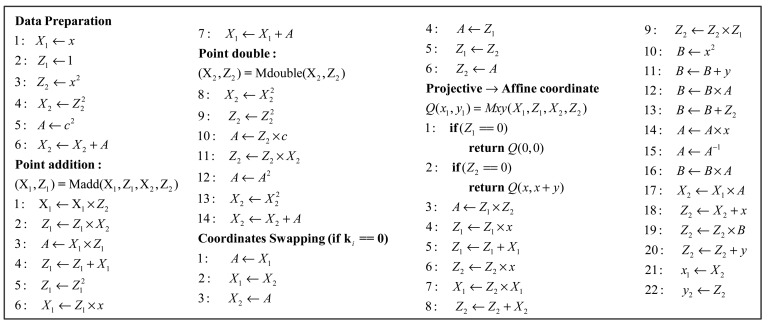
Routines implemented in the controller.

The ECP command controller, as shown in Figure 7, executes data preparation, point addition, point doubling, and the Mxy according to Algorithm 3. The data preparation is carried out simply by transferring (x,y) into (*X_1_, Z_1_, X_2_, Z_2_*), from the affine coordinates to the López-Dahab projective coordinates. The Mxy part involves the final calculations for converting projective coordinates to affine coordinates. The routines implemented in the ECP command controller are collected in Figure 8.

According to part 3 of Algorithm 3, the ECP command controller takes in the most significant bit (MSB) of the shift register *k* from register array. No matter if *k_i_* = 0 or *k_i_* = 1, a point addition and a point doubling are performed in one iterative calculation. Figure 8 shows that five registers are needed to store temporary variables in the calculation of point addition and point doubling. The base point coordinate *x* and EC parameter *c* will be loaded into the constant register *c* from the external ROM when they are needed.

In the iterative calculation of the part 3 of Algorithm 3, when *k_i_* = 1, point addition and point doubling are performed as follows:

(9) $$A\leftarrow X_{1}Z_{2}Z_{1}X_{2},Z_{1}\leftarrow {\left(X_{1}Z_{2}+Z_{1}X_{2}\right)}^{2},X_{1}\leftarrow xZ_{1}+A$$

(10) $$A\leftarrow c^{2}Z_{2}^{4},Z_{2}\leftarrow X_{2}^{2}Z_{2}^{2},X_{2}\leftarrow X_{2}^{4}+A$$

when *k_i_* = 1, at the beginning of point addition, *X_1_*, *Z_1_*, *X_2_*, *Z_2_* will be stored in Reg_A, Reg_B, Reg_C Reg_D respectively. The result of point addition will be stored in *X_1_*, *Z_1_*. In other words, the value of Reg_A and Reg_B will be updated with the new value of *X_1_* and *Z_1_*. Reg_C and Reg_D will keep the previous value of *X_2_* and *Z_2_*. As soon as the calculation of point addition is finished, the calculation of point doubling will begin. The inputs to this operation are *X_2_*, *Z_2_*, and EC parameter *c*. By the way of circular shift, register array will provide the right operand to MALU, and the previous value of *X_1_*, *Z_1_* will be kept in the circular shift. The outputs to point doubling are saved in *X_2_*, *Z_2_*. Therefore, the values in Reg_A, Reg_B, Reg_C and Reg_D will be updated with the new value of *X_1_*, *Z_1_*, *X_2_*, *Z_2_*, respectively, after the calculation of one point addition and point doubling. Four registers are utilized to store the value of *X_1_*, *Z_1_*, *X_2_*, *Z_2_*, and another one is used to store the value of *A*.

When *k_i_* = 0, point addition and point doubling are performed as follows:

(11) $$A\leftarrow X_{1}Z_{2}Z_{1}X_{2},Z_{2}\leftarrow {\left(X_{1}Z_{2}+Z_{1}X_{2}\right)}^{2},X_{2}\leftarrow xZ_{2}+A$$

(12) $$A\leftarrow c^{2}Z_{1}^{4},Z_{1}\leftarrow X_{1}^{2}Z_{1}^{2},X_{1}\leftarrow X_{1}^{4}+A$$

According to Equations (9) and (11), when *k_i_* changes from 1 to 0, the terms *X_1_Z_2_Z_1_X_2_* and *X_1_Z_2_* + *Z_1_X_2_* will remain unchanged. When *k_i_* = 0, before the calculation of point addition and point doubling, *X_1_* and *Z_1_* will be swapped with *X_2_* and *Z_2_*. Therefore, *X_1_*, *Z_1_*, *X_2_*, *Z_2_* will be stored in Reg_C, Reg_D, Reg_A, Reg_B respectively. The outputs to point addition are saved in *X_2_*, *Z_2_* (Reg_A, Reg_B) and the outputs to point doubling are saved in *X_1_*, *Z_1_* (Reg_C, Reg_D). As the updated result value of *X_1_*, *Z_1_*, *X_2_*, *Z_2_* are stored in Reg_C, Reg_D, Reg_A, Reg_B respectively, a coordinate swapping is still needed to swap *X_1_*, *Z_1_* with *X_2_*, *Z_2_*, in the end of point doubling. Therefore, the final results of *X_1_*, *Z_1_*, *X_2_*, *Z_2_* are stored in Reg_A, Reg_B, Reg_C, Reg_D, respectively.

Consequently, the point addition and point doubling can be repeated in the iterative calculation in the part 3 of Algorithm 3 without the involvement of *k_i_* bits. However, when *k_i_* = 0, it needs extra coordinates swapping operations. By the way of coordinates swapping, it is easy to implement the controller and decrease the complexity. In addition, it can shorten the time of iterative calculation effectively.

As Figure 8 shows, in data preparation, it needs three squaring and one field addition. Six multiplications, five squaring and three field additions are performed in the calculation of one point addition and point doubling. In the phase of converting projective coordinates to affine coordinates, including an inversion operation, there are total of 19 multiplications, 163 squaring and 6 field additions. Assuming *H*(*k*) = 163, the hamming weight of private *k*, point multiplication will take approximately 163.9 K clock cycles without clock cycles of controlling. When the base point coordinate *P*(*x, y*) or EC parameter *c* is utilized as one operand, the constant register requires a read operation, and the operation will take 21 clock cycles. The final simulation result shows that point multiplication will take 176.7 K clock cycles based on our whole design scheme.

## 7. Low Power Strategies

As the energy provided for passive RFID tag comes from radio frequency signals, low power consumption is an essential requirement for passive RFID tag chip. The operation distance of RFID systems depends on the maximum of the dynamic power of tag chip. Clock-gating and asynchronous counters are adopted in the design of our ECC processor to minimize power consumption.

Clock gating is a popular technique used in many synchronous circuits for reducing dynamic power dissipation [24]. Clock gating saves power by adding more logic to a circuit to prune the clock tree. Pruning the clock disables portions of the circuitry so that the flip-flops in them do not have to switch states. The used clock gating cells come from UMC standard cell library in 0.13 μm CMOS technology.

**Figure 9.**
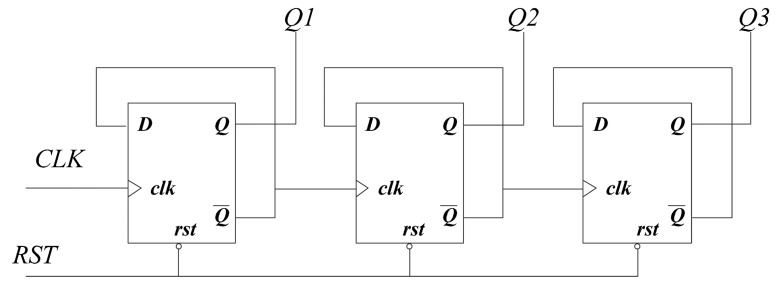
Asynchronous counter.

Many counters are used in the design of ECC processor. Using synchronous counter might bring in large power consumption, since every bit of the registers would be triggered on each of the clock edges. For asynchronous counter, as shown in Figure 9, only the first flip-flop would be triggered by *clk*. The subsequent flip-flops are triggered by the former flip-flops. Therefore, the unnecessary switches in registers can be minimized as well as reducing the dynamic power. As the ECC processor for RFID tag works at a low clock frequency, there is no need to use synchronous counters. Compared to synchronous counter, asynchronous counters with more than 4-bit flip-flops structure can reduce the power consumption by 50% at least [25].

## 8. Result and Comparison

### 8.1. Result Analysis

The proposed design has been conducted in Verilog HDL. We synthesized our processor using a low leakage power library of UMC 0.13 μm (fsc0l_d_generic_core_tt1p2v25c.db). For synthesis, we used the EDA tool of Design Complier. Gate equivalent (GE) can provide meaningful comparisons of area among different technologies. The area of two inputs NAND is 5.12 μm^2^ in *fsc0l* lib. Table 1 shows the gate count of every component of our ECP. The controller and the MALU have the area of 918 GE and 3591 GE respectively. The register array (9342 GE) dominates the gate area and occupies 67% area of the whole design.

**Figure 10.**
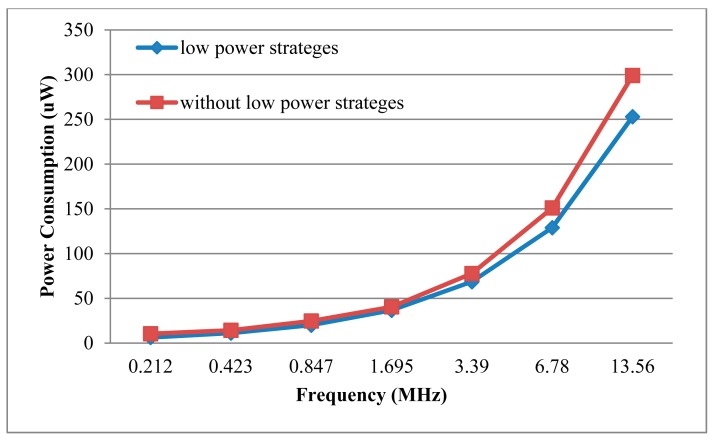
Power consumption under different work frequencies.

**Figure 11.**
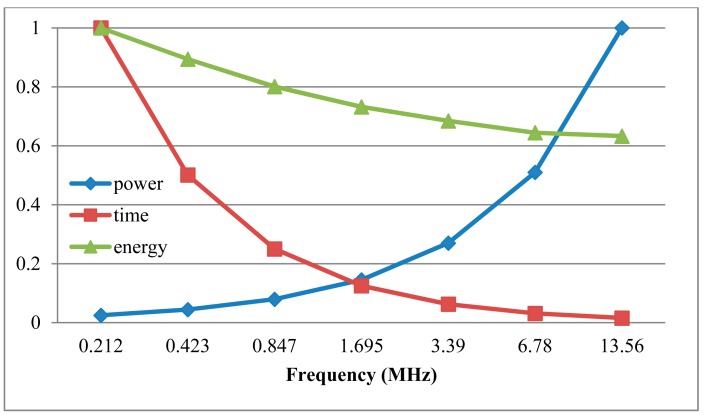
Normalized time, power and energy for one scalar point multiplication under different work frequencies.

Power consumption is one of the most important factors for passive RFID tag chip. We used EDA tools of VCS and Power Compiler to get the estimation of average power at the gate level. First, the Value Change Dump (VCD) files were generated in VCS. Then, VCD files were translated to Switching Activity Interchange Format (SAIF) files, which were used in Power Complier to get average power consumptions. The carrier frequency of ISO/IEC 14443 standard is 13.56 MHz [26], so the source clock frequency provided by AFE is 13.56 MHz.When the ECP is applied to the RFID tag chip, the ECP will be under the control of the baseband. The work clock of ECP comes from the baseband. The source clock may be divided by 2*^n^*, and the divided clock will be provided for the ECP, where *n* = 0, 1, 2, 3, 4, 5, 6. Figure 10 and Table 2 show the ECP's power consumption, time and energy for one scalar point multiplication at different work frequencies and the power consumption can be effectively reduced by decreasing the operation frequency. Because of the adoption of low power strategies, such as clock gating and asynchronous counter, it can save about 15% power consumption at the same work clock frequency. In order to compare the frequency power benefits, Figure 11 shows the normalized time, power and energy for one scalar point multiplication under different work frequencies.

The simulation result of a certain scalar point multiplication demonstrates that the time needed for one calculation is 176.7 K clock cycles. Taking the ISO/IEC 14443 standard as an example [26], the AFE will extract a clock with the frequency of 13.56 MHz, so the total time consumption is nearly 13.1 ms.

**Figure 12.**
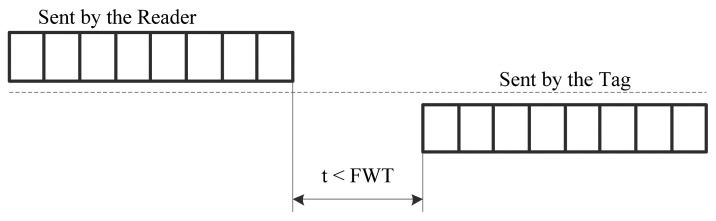
Frame waiting time.

As the ISO 14443 standard specified, the frame waiting time (FWT) defines the maximum time for a RFID tag to start its response frame after the end of a reader frame, as shown in Figure 12. The FWT is calculated by the following formula:

(13) $$\text{FWT}=(256\times 16/fc)\times 2^{FWI}$$

where *fc* is the carrier frequency and where FWI is the frame waiting time integer. FWI is coded in the range from 0 to 14. The default value of FWI is 4, which means that FWT = 4.8 ms by default. However, when the tag needs more time to process the information it receives, such as the ECC operations, it can impose the reader to increase the FWI up to 6, which corresponds to FWT = 19.3 ms. In fact, the total transaction time of most RFID applications is required less than 300 ms. For example, a validation process at a gate should take less than 300 ms usually [27]. The Hong Kong mass transit smart card takes about 140–300 ms for one transaction [28]. The smaller FWT can reduce the customers' waiting time effectively. Our design requires 13.1 ms for one ECC operation at 13.56 MHz. Therefore, it is compatible with the ISO 14443 standard and most RFID applications, such as mass transit smart card.

**Figure 13.**
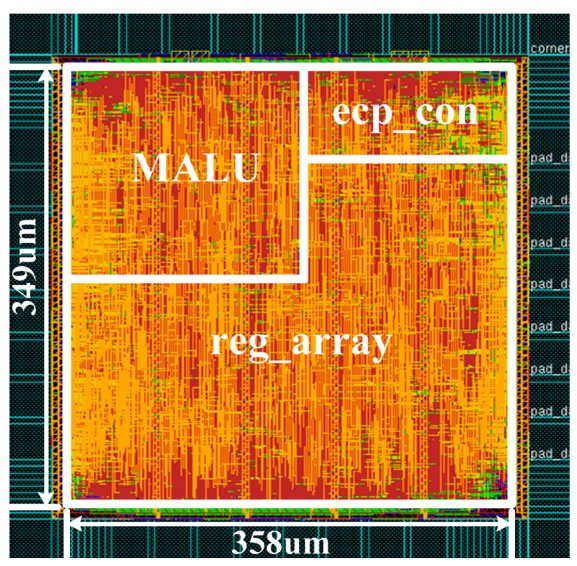
Final Layout Result.

Both the power consumption and the calculation time prove that our proposed ECP can meet the demands of real-time and resource-constraint for the applications of RFID tag chips. The layout of the ECP is shown in Figure 13. The largest module is the register array, followed by the MALU module, and the ECP command controller is the smallest module. The overall area of the ECP processor is 349 μm × 358 μm.

### 8.2. Comparisons

Table 3 shows the comparison with the related works. The ECP can work at different clock frequencies according to the real-time and power requirements of different RFID systems. The number of clock cycles for one scalar point multiplication is 176.7 K and it is much less than the design of [10–13].

The MALU in the design of [10] has an 8-bit adder and an 8-bit multiplier. Therefore, the squaring operation uses the same logic as the multiplication, hence, each squaring requires *m* clock cycles. In our design, there is a specialized squarer unit and the squaring operation requires only one clock cycle. Moreover, the design of [10] assumes that the coordinate conversion to the affine coordinate system and the calculation of *Y*-coordinate value are performed on a reader or back-end system, hence, the inversion operation has not been implemented in [10]. If the inversion is performed in the design of [10], another 163-bit register should be added. The area of one bit register composed of D-type flip-flops is about 8 GE in UMC 0.13 μm technology. In fact, the coordinate conversion is necessary in a complete ECP of the RFID tag chip for encryption and decryption applications, such as ECDSA [29]. Due to the adoption of the DJ method, one 163-bit register is saved in the inversion operation of our design. As a result, the number of 163-bit register is the same as [10], but the coordinate conversion is implemented in our scheme. The design of [10] can acquire the trades-offs between the gate area and the number of clock cycles depending on the digit sizes, but the time cost for one scalar point multiplication is about 240 ms, it can hardly satisfy the transaction time requirements of some RFID systems.

The literature [12] has adopted the same method and structure as [10] does. The ECs parameters are fixed that can be very helpful to reduce the area, but it is not compatible in different RFID systems. In our design, the register *c* of register array can load in different ECs parameters. Moreover, the EC in [12] is a kind of binary Edwards curve, which is a special elliptic curve. The binary Edwards curves have not been recommended by NIST standards [30] and are not widely used in cryptosystems. The Koblitz curve is widely used and is adopted in our design.

In [11], the ECP has been implemented in an affine coordinate system, with the disadvantage that it requires one inversion to be computed in each iteration. As a result, it needs much more clock cycles than our work for one scalar point multiplication. Moreover, the ECs parameter *c* in [11] is fixed. Hence, the design of [11] cannot be used in other Koblitz curves when the parameter *c* is changed. In [11], it does not achieve a much better result than ours or other works. Finally, in [13], the scalar point multiplication is done in 297 K clock cycles and the area is 13.2 K GE. The gate area of our design is slightly larger than [13], but the number of clock cycles is much less than [13].

Due to the adoption of clock gating and asynchronous counter, the power consumption is smaller than all the other designs at the same clock frequency. With the power and the time to finish one scalar point multiplication, we can get the energy consumption demonstrating that our design can satisfy the low-power requirement of RFID tag chips. The low number of clock cycles needed for one scalar point multiplication makes it suitable for real-time applications of RFID tag chips. Although the gate area of our design is slightly larger than [12,13], our design achieves a good trade-off between the real-time requirement and the constrained resource.

## 9. Conclusions

A design of Elliptic Curve Cryptography processor based on Koblitz curves for the RFID tag chip is presented in this paper. We employ Montgomery Ladder algorithm for scalar point multiplication which is the underlying operation in the elliptic curve cryptography. A new DJ method is exploited for inversion operation and it requires fewer registers to store variables than the traditional methods. A preferable MALU for our ECP design is presented in detail. In the iterative calculation of point addition and point doubling, a new coordinates swapping method is proposed. It can reduce the complexity of the ECP command controller and shorten the time of iterative calculation effectively. The modified circular shift register file is introduced to reduce the complexity of system. Some low power strategies, such as clock gating and asynchronous counter are adopted, saving about 15% power consumption.

Our design was synthesized with UMC 0.13 μm CMOS technology at different frequencies. Compared to other reported results, our architecture acquires good trade-offs of clock cycle number, gate area, power consumption and energy. On the aspects of power consumption and time cost, our design shows better performance. According to the implementation results, the ECP area is 0.12 mm^2^, and the power consumption can be reduced to 20.1 μW at the clock frequency of 847.5 KHz, which can meet the demands of real-time, low-power, and resource-constraints for the applications of RFID tag chips and WSNs.

## Figures and Tables

**Table 1.** Gate area of individual processor components.

	**Ecp_con**	**MALU**	**Reg_array**
Cell Area (μm^2^)	4702.7	18385.9	47831.1
Area (Gates)	918	3591	9342

**Table 2.** Power, time and energy consumptions under different work frequencies.

Freq (KHz)	13,560	6780	3390	1695	847.5	423	212
Power (μW)	253	129	68.6	36.7	20.1	11.2	6.27
Time (ms)	13.1	26.1	52.2	104.5	208.5	417.7	833.5
Energy (μJ)	3.31	3.37	3.58	3.83	4.19	4.68	5.23

**Table 3.** Comparison with related works.

**Ref.**	**Freq.(KHz)**	**Power (μW)**	**Cycles**	**Time (ms)**	**ECP Area (gates)**	**Core Size (μm^2^)**	Energy * (μJ)	**Technology**
This Work	13,560	253	176.7 K	13.1	13.8 K	124,942	3.3	UMC 0.13 μm
	1,690	36.7		104.5			3.8	
	847.5	20.1		208.5			4.2	
	423	11.2		417.7			4.7	

[10]	1,130	36.63	275.8 K	244.08	12.5 K	N.A.	8.9	UMC 0.13 μm
	590	21.55	144.8 K	245.49	14.1 K		5.3	
	411	15.75	101.2 K	246.19	14.7 K		3.9	
	323	12.08	78.5 K	243.17	15.4 K		2.9	

[11]	13,560	N.A.	376.9 K	31.8	15 K	N.A.	N.A.	AMI 0.35 μm

[12]	400	7.3	219.1 K	547.87	11.7 K	N.A.	3.9	UMC 0.13 μm

[13]	847.5	83		350.4		219,897	29.1	UMC 0.18 μm
	106	10.8	297 K	2801.9	13.2 K		30.2	
	106	54.7		2801.9		N.A.	153.2	AMS 0.35 μm

* Energy consumption for one scalar point multiplication.
